# Feasibility of a Modified Wake‐Up Test Without Intraoperative Neurophysiological Monitoring in Scoliosis Surgery: A Case Series

**DOI:** 10.1155/cria/8811380

**Published:** 2026-05-27

**Authors:** Luan D Ta, Hau V Nguyen, Chinh V Vu, Duc T. Lam

**Affiliations:** ^1^ Head of the Department of Anesthesia and Intensive Care, Faculty of Medicine, Nguyen Tat Thanh University, Ho Chi Minh City, Vietnam, ntt.edu.vn; ^2^ Ho Chi Minh City Orthopedic and Rehabilitation Hospital, Ho Chi Minh City, Vietnam; ^3^ Hospital for Traumatology and Orthopedic, Ho Chi Minh City, Vietnam; ^4^ Faculty of Medical Laboratory Technology, Nguyen Tat Thanh University, Ho Chi Minh City, Vietnam, ntt.edu.vn

**Keywords:** anesthesia, muscle relaxants, scoliosis surgery, spinal cord function, sugammadex, wake-up test

## Abstract

The wake‐up test (WKT) is an important intraoperative method for assessing spinal cord function during scoliosis surgery, particularly in settings where intraoperative neurophysiological monitoring (IONM) is unavailable. This short case series describes seven patients undergoing scoliosis surgery with a modified wake‐up test (mWKT). Sugammadex was used to reverse neuromuscular blockade to facilitate motor assessment. All patients were able to follow commands and demonstrate lower‐limb movement during the test. The duration of mWKT ranged from 15 to 35 min and decreased with increasing procedural familiarity. One patient required naloxone due to delayed recovery. Importantly, no episodes of bradycardia or clinically significant hemodynamic instability were observed following sugammadex administration. Only one patient reported limited postoperative recall. This case series suggests that the WKT remains a feasible and safe alternative when IONM is unavailable. Sugammadex may facilitate more efficient neuromuscular recovery and improve procedural control.

## 1. Introduction

Intraoperative neurophysiological monitoring (IONM) plays a vital role in spinal surgeries, particularly in scoliosis correction, where the spinal cord is at risk of injury from direct manipulation or tension‐induced ischemia. IONM provides real‐time feedback to ensure the functional integrity of the spinal cord during surgery, thereby reducing the risk of neurological damage [1–3]. A key component of this monitoring is the wake‐up test (WKT), performed at the end of surgery to assess motor function in the lower extremities. This test helps detect any immediate spinal cord injury before the incision is closed, allowing for corrective actions if necessary [4]. The effectiveness of IONM, particularly the WKT, has been shown to reduce severe neurological complications in scoliosis surgery, highlighting the importance of such monitoring in improving patient outcomes [4] (Paunikar et al., 2023).

Numerous studies have investigated anesthetic techniques for assessing spinal cord function during surgeries [5]. Opioids and intravenous anesthetics are commonly preferred over inhalational agents, with muscle relaxants minimized, particularly during intubation [6, 7]. When spinal cord function is assessed, such as in nervous system surgeries, anesthetics are adjusted for gradual awakening, known as the WKT. This method allows clinicians to evaluate spinal cord integrity by assessing responsiveness after anesthesia [4]. However, it does not provide detailed localization of nerve damage and carries the risk of distressing memories [8].

In recent years, IONM has emerged as a valuable tool in surgical procedures, offering an alternative to the traditional WKT. The primary advantage of IONM is its ability to detect neurological complications in real time, allowing for immediate identification of damage and enabling surgeons to intervene swiftly to mitigate adverse outcomes [9]. This capability is particularly critical during high‐risk surgeries, where the early detection of neurological impairment can significantly improve patient outcomes. However, the widespread adoption of IONM faces several challenges. The need to minimize or avoid the use of inhalational anesthetics and neuromuscular blocking agents in prolonged surgeries presents a significant challenge for anesthesiologists when applying IONM, as these drugs can interfere with electrophysiological signals [10]. Additionally, the need for specialized personnel to operate IONM equipment further limits its accessibility in many medical settings. The scarcity of electrodes and the limited availability of IONM machines, compounded by the high costs associated with this technology, are significant barriers to its broader implementation. Moreover, concerns about the reliability of IONM systems, particularly the risk of false negatives, have led some surgeons to remain cautious about fully replacing the WKT with IONM [11].

In cases where IONM is unavailable or not feasible, WKT remains a reliable alternative for assessing neurological function during surgery. However, this test is not without its challenges, including the need for meticulous anesthesia management during prolonged procedures. Recent advancements, such as the introduction of sugammadex, a next‐generation muscle relaxant, have significantly improved the efficiency and safety of the WKT. Sugammadex allows for the rapid reversal of muscle relaxation, even in deep relaxation states, facilitating a quicker and more efficient WKT [12]. This advancement has made anesthesia and muscle relaxation safer and more manageable, thereby enhancing the overall feasibility and effectiveness of the WKT when IONM is not an option.

This report is presented as a short case series to highlight practical clinical insights from seven patients undergoing scoliosis surgery with a modified wake‐up test (mWKT) in a resource‐limited setting.

## 2. Case Presentation

This report is presented as a short case series describing seven patients who underwent scoliosis surgery with an intraoperative WKT. Seven patients undergoing scoliosis surgery with a planned WKT were included. As all patients in this case series were minors, written informed consent was obtained from their legal guardians, and the study was conducted in accordance with institutional ethical standards.

Patient characteristics and operative details are summarized in Table 1.

 Case 1: A 25‐year‐old female (ASA II) underwent scoliosis correction under balanced anesthesia with sevoflurane, fentanyl, and rocuronium. Sugammadex (200 mg total) was administered prior to mWKT. The patient followed verbal commands and lifted both legs within 35 min. Hemodynamic parameters remained stable, with no bradycardia observed.
 Case 2: A 26‐year‐old female (ASA II) received similar anesthetic management. After sugammadex (100 mg), the patient successfully lifted both legs within 25–28 min. No complications occurred.
 Case 3: A 15‐year‐old female (ASA I) underwent surgery without sugammadex reversal. The patient demonstrated adequate responsiveness and lifted both legs after approximately 30 min.
 Case 4: A 16‐year‐old male (ASA II) underwent total intravenous anesthesia with propofol and sufentanil. Due to delayed recovery, naloxone was administered. The patient was eventually able to lift both legs, but the WKT duration was prolonged to 45 min.
 Case 5: A 16‐year‐old female (ASA I) received sugammadex (100 mg) and demonstrated rapid recovery, lifting both legs within 15–17 min.
 Case 6: A 15‐year‐old female (ASA II) also received sugammadex (100 mg) and showed similar rapid recovery, with leg movement observed within 15–16 min.
 Case 7: An 11‐year‐old female (ASA II) did not receive sugammadex but demonstrated spontaneous recovery and was able to lift both legs within 10–14 min.

**TABLE 1:** Characteristics of surgical patients.

Cases	Weight/height (kg/cm)	BMI (kg/m^2^)	ASA	Anesthesia time (hr/min)	Surgery time (hr/min)	Anesthetic drugs
1. Female, 25 yrs	49/158	19.6	II	6:00	5:00	Sevoflurane + Fentanyl + Rocuronium
2. Female, 26 yrs	45/155	18.7	II	6:00	5:00	Sevoflurane + Fentanyl + Rocuronium
3. Female, 15 yrs	44/156	18.1	I	5:05	4:00	Sevoflurane + Fentanyl + Rocuronium
4. Male, 16 yrs	63/165	23.1	II	8:30	7:40	TIVA Propofol + Sufentanil + Succinylcholine (with IONM)
5. Female, 16 yrs	46/165	16.89	I	4:10	3:25	Sevoflurane + Fentanyl + Rocuronium
6. Female, 15 yrs	40/148	18.2	II	5:45	4:50	Sevoflurane + Fentanyl + Rocuronium
7. Female, 11 yrs	41/148	18.41	II	8:30	6:00	Sevoflurane + Fentanyl + Rocuronium

*Note:* BMI (kg/m^2^): Body Mass Index; ASA: American Society of Anesthesiologists Physical Status.

Overall, the mWKT was successfully completed in all patients. The time required ranged from 15 to 35 min in most cases, with a longer duration observed in one patient requiring naloxone. Postoperative recall was uncommon, with only one patient reporting limited awareness. Importantly, no episodes of bradycardia or clinically significant hemodynamic instability were observed following sugammadex boluses in any of the cases.

Detailed information on anesthetic management and WKT performance is presented in Table 2.

**TABLE 2:** Modified wake‐up test (mWKT) mPRST score during WKT.

Cases	Anesthesia time before mWKT (hr/min)	Sugammadex	Propofol	Fentanyl	Leg lift time (min)
Case 1	4/10	100 + 100	No	No	35 (Right), 32 (Left)
Case 2	4/10	100	No	No	25 (Right), 28 (Left)
Case 3	3/25	No	No	No	30 (Right), 30 (Left)
Case 4	5/45	Naloxone 4 mg	2 mg/kg/h	No	45 (Right), 45 (Left)
Case 5	3/55	100	No	No	15 (Right), 17 (Left)
Case 6	4/50	100	No	No	15 (Right), 16 (Left)
Case 7	5/30	No	No	No	10 (Right), 14 (Left)

**FIGURE 1:**
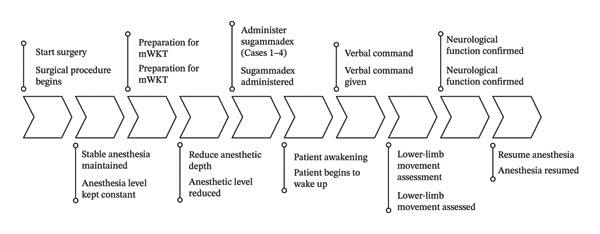
Workflow of the modified wake‐up test (mWKT) applied in Cases 1–4. The process includes stepwise anesthetic reduction, administration of sugammadex for neuromuscular reversal, and assessment of voluntary lower‐limb movement to evaluate intraoperative neurological function.

The workflow of the mWKT applied in representative cases is illustrated in Figure 1.

## 3. Discussion

This short case series highlights several key clinical insights. First, the WKT remains a feasible and reliable method for intraoperative neurological assessment when IONM is unavailable. All seven patients were able to demonstrate purposeful lower‐limb movement, supporting the practical applicability of this technique in resource‐limited settings.

Second, sugammadex appears to facilitate more efficient neuromuscular recovery and may shorten the time required to perform the WKT. In our series, patients receiving sugammadex generally demonstrated faster recovery compared with those who did not. However, variability in wake‐up time was observed, likely reflecting differences in anesthetic techniques, duration of surgery, and individual patient factors rather than neuromuscular blockade alone. Importantly, no bradycardic responses or clinically significant hemodynamic instability were observed following sugammadex administration, supporting its safety profile in this context.

Third, intraoperative recall was uncommon, with only one patient reporting limited awareness. This finding suggests that the WKT can be performed without significant patient distress when appropriate anesthetic management is applied.

The absence of quantitative neuromuscular monitoring and processed EEG represents a limitation, as it limits precise differentiation between delayed awakening and residual neuromuscular blockade. Therefore, findings should be interpreted descriptively.

Although IONM is widely used in high‐resource settings, the WKT remains a practical and clinically meaningful alternative. This case series emphasizes its continued relevance and provides practical insights for clinicians working in environments where advanced monitoring is not available.

## 4. Conclusion

There are multiple approaches to conducting the mWKT depending on available equipment, clinician preference, and patient factors. In this series, mWKT was feasible under a balanced anesthetic technique and could be facilitated intraoperatively with sugammadex and small doses of propofol when needed. Sugammadex appeared to shorten the interval to perform mWKT, and intraoperative recall was uncommon. Importantly, although mPRST was used when processed EEG was unavailable, physiologic signs alone are not reliable for assessing anesthetic depth. Processed EEG monitoring (BIS/entropy) should be preferred whenever available, with mPRST serving only as a fallback in resource‐limited settings. Future studies incorporating pEEG and quantitative neuromuscular monitoring are warranted to standardize WKT protocols and outcomes.

## Author Contributions

Luan D Ta, Duc T. Lam: conceptualization, methodology, formal analysis, investigation, data curation, visualization, writing–original draft, writing–review and editing, and project administration.

Duc T. Lam: investigation, data curation, validation, and writing–review and editing.

Luan D Ta: investigation, resources, validation, and writing–review and editing.

Luan D Ta: data curation and investigation.

Luan D Ta: supervision, methodology, validation, and writing–review and editing.

Chinh V Vu: investigation, resources, validation, and writing–review and editing.

Hau V Nguyen: software, formal analysis, visualization, methodology, and writing–review and editing.

## Funding

This research received no specific grant from any funding agency in the public, commercial, or not‐for‐profit sectors.

## Disclosure

All authors have read and approved the final manuscript.

## Ethics Statement

This work was approved by Orthopedics and Rehabilitation (1A Hospital, Viet Nam), approval no. 132/QĐ‐HĐĐĐ. Written informed consent was obtained from all participants or their legal guardians. The study was conducted in accordance with recognized ethical principles, including the Declaration of Helsinki.

## Consent

Written informed consent was obtained from the patient for publication of this report and accompanying images.

## Conflicts of Interest

The authors declare no conflicts of interest.

## Supporting Information

Additional supporting information can be found online in the Supporting Information section.

## Supporting information


**Supporting Information** Appendix S1. Modified PRST (mPRST) Score.

## Data Availability

The data supporting the findings of this study are available from the corresponding author upon reasonable request. No publicly available datasets were generated or analyzed during the current study.
