# Supplementary material for: Feasibility of a Modified Wake‐Up Test Without Intraoperative Neurophysiological Monitoring in Scoliosis Surgery: A Case Series
Source: Case Rep Anesthesiol. 2026 May 27;2026:8811380. doi: 10.1155/cria/8811380 (PMC13215055; doi:10.1155/cria/8811380)
Supplement: Supplementary file 1 — Supporting Information Appendix S1. Modified PRST (mPRST) Score. [file CRIA-2026-8811380-s001.docx]

**Supplementary Material**

**Appendix S1. Modified PRST (mPRST) Score**

The PRST score is a clinical scoring system based on changes in pulse rate (P), systolic blood pressure (S), sweating (R), and tearing (T). It was historically used to describe physiological responses to surgical stimulation during general anesthesia. In contemporary practice, the PRST score has largely been replaced by processed electroencephalographic monitoring and is not considered a validated measure of anesthetic depth or awareness.

In this case series, a modified PRST score (mPRST) was used as a descriptive physiological observation tool during the modified wake-up test (mWKT) when processed EEG monitoring was unavailable. The mPRST score was not used to guide anesthetic management and was not intended to assess pain, depth of anesthesia, or consciousness.

**Score Components**

The mPRST score consisted of four observable parameters:

- Pulse rate (P): Increase in heart rate relative to baseline
- Systolic blood pressure (S): Increase in systolic arterial pressure relative to baseline
- Sweating (R): Presence of visible diaphoresis
- Tearing (T): Presence of lacrimation

Each parameter was assigned a value of 0 (absent) or 1 (present), yielding a total score ranging from 0 to 4.

**Timing of Assessment**

The mPRST score was recorded at two predefined time points:

1. Before mWKT: Immediately prior to anesthetic reduction in preparation for the modified wake-up test
2. During mWKT: At the time of peak observable physiological response during patient responsiveness

Values reported in the tables represent single peak clinical measurements and were not averaged over time.

**Interpretation**

The mPRST score was used to document observable physiological changes during anesthetic emergence. It was interpreted descriptively and not as a surrogate for anesthetic depth, analgesic adequacy, or awareness. The absence of sweating and tearing across cases reflects the limited sensitivity of these parameters under modern anesthetic conditions.

The use of the mPRST score in this report reflects pragmatic clinical observation in a setting without advanced monitoring and does not imply endorsement of the PRST score as a contemporary anesthetic monitoring tool.
